# Influence of typical handball characteristics on upper body posture and postural control in male handball players

**DOI:** 10.1186/s13102-020-0156-2

**Published:** 2020-03-02

**Authors:** D. Ohlendorf, S. Salzer, R. Haensel, J. Rey, L. Maltry, F. Holzgreve, J. Lampe, E. M. Wanke, D. A. Groneberg

**Affiliations:** 1 Institute of Occupational Medicine, Social Medicine and Environmental Medicine, Goethe-University, Theodor-Stern-Kai 7, 60590 Frankfurt am Main, Germany; 2 Institute of Biostatistics and Mathematical Modeling, Goethe-University, Frankfurt/Main, Theodor-Stern-Kai 7, 60590 Frankfurt/Main, Germany

**Keywords:** BMI, Playing years, Playing position, Throwing arm, Handball

## Abstract

**Background:**

Well defined constitutional parameters support the physical fatigue resistance in handball to maintain the performance level for the majority of actions. Ideal constitutional conditions are necessary to achieve these physiological advantages in handball. But limited knowledge exists about the upper body posture or the postural control in correlation to the Body Mass Index (BMI), playing years, playing position and throwing arm in professional male handball.

**Methods:**

Ninety-one male handball players participate (24.1 ± 5.9 years; playing experience 16.6 ± 5.7 years). A three-dimensional back scanner and a pressure measuring plate were used.

**Results:**

Correlations between BMI and upper body posture and postural control were not significant. Same counts for the comparison between the left and right throwing arm according to upper body posture and postural control (*p* ≥ 0.05). Correlations between the years of playing can be found at pelvis height (*p* ≤ 0.04) and for the length of the Center of Pressure (CoP) (*p* ≤ 0.01). Wing players are 6.5–8.5 cm smaller. The playing position is independently of BMI, age or upper body posture (*p* ≥ 0.05). Backcourt players have a higher load of the left and a lower load of the right foot compared to wing players (*p* ≤ 0.001). Left-right comparison (*p* ≤ 0.001/ 0.01) can be seen in pivot player (covered area), backcourt player (weight distribution left/right [rear] foot), wing player (weight and force distribution left/right foot, covered area).

**Conclusion:**

Goalkeeper, Backcourt and pivot players are taller and heavier than wing players. These physiological demands are not detectable in the upper body posture and slightly in postural control. Wing players have the most asymmetric load distribution and the longest length of CoP. Since goalkeepers do not differ from pivot or backcourt players, this can be lead back to the same training.

## Background

Especially at the highest level of performance sports, slightest physical differences can make the difference between victory and defeat. In complex sports, sports motor abilities and skills combined with anthropometric parameters are among the decisive performance factors. One sport, in which such parameters are important, is the physical Olympic contact sport of handball. Due to its multitude of tackles (hard body confrontations) in offence and defense within a 60-min, high-intense technical play with strength-related playing actions and high-intensity changes, the requirement for a good physical constitution becomes clear. A good physical constitution is reflected among other things in the body size and the body weight [1, 2].

Literature shows various differences in physical constitution and performance capability between amateur handball players and elite handball players. Top elite male handball players are taller and heavier but also faster in the sprint than the amateur handball players [3–5]. Gorostiaga et al. [4] confirmed that elite handball players have a higher efficiency in throwing velocity as well as higher absolute maximal strength and muscle power than amateur handball players. They attribute the higher efficiency in handball throwing velocity to the better power output capabilities of the upper and lower extremities as well as to a greater free fatty mass. Male handball players with a body height above the average have a slightly larger kyphosis angle than smaller players (same trainings level) [6].

Furthermore, different physiological demands for different playing positions are required in handball: backcourt players or pivot players are taller and heavier and show higher intensities (max. Heart rate) than wing players (left/right side of the game) [7–10].

Well defined constitutional parameters support the physical fatigue resistance in handball to maintain the performance level for the majority of actions [11]. Improvements in the performance capability of handball players can be attained through specific training interventions like a systematic throwing velocity training, e.g. with medicine balls, from different throwing positions. Here, a significant improvement in throwing velocity (4.5%) can be achieved [12, 13]. Manchado et al. [12] explain that an increase in the strength and stability of the lumbo-pelvic region can contribute to an improvement in the kinetic chain of throwing in handball. A significant increase in oxygen uptake, offense time, defense time, fast break time, and jump height in male elite handball players proved Wagner et al. [14] by a 1-year specific physical training program in addition to a normal weekly training (team handball techniques and tactics).

To achieve these physiological advantages in handball, ideal constitutional conditions are necessary, such as a harmoniously pronounced musculoskeletal system or a well-developed postural control as a complex feedback-dependent system by various sensory inputs from visual, vestibular and somatosensory receptors [15–17]. So far, it is only known that goalkeepers of the Polish National Junior Handball Team have a very good postural stability [18]. Basically, a harmoniously pronounced musculoskeletal system is recommended, whereby it should be considered that a certain muscular asymmetry is also the result of handiness and intensive training. Too pronounced asymmetries can be a predictor of muscular imbalances and consequently musculoskeletal complaints.

Body anthropometry seems to have an important influence on playing performance and it is related to the playing position. Nevertheless, limited knowledge exists about the upper body posture or the postural control (body weight distribution and postural sagittal/frontal sway) in correlation to the BMI, playing years, playing position and throwing arm in professional male team handball player. The working hypotheses of this study are as follows:

1. Playing position-specific characteristics of the upper body posture in male handball players can be recognized as a consequence of body height, weight and BMI.
2. The BMI has no influence on the upper body posture or the frontal/sagittal body sway in male handball players.
3. An increase in the years of playing can be seen in an asymmetric upper body posture and in an asymmetrical shift in terms of the percentage body weight distribution in male handball players.
4. The influence of the throwing arm can be seen in asymmetrical muscular expressions of the shoulder region in male handball players.
5. Handball male players (according to their playing position) have an asymmetric upper body posture which corresponds to the percentage body weight distribution while standing.

## Methods

### Subjects

Ninety healthy, randomly chosen male handball players took part in this study, age ranging from 17 to 44 years (average age 24.3 ± 5.8 years). Participants had a playing experience from 10 to 36 years (16.6 ± 5.7 years).

All participants came from four different teams of five different clubs, with players actively playing in the 1st “Handball Bundesliga” (HBL) to “Bezirksklasse”. Depending on the club, the players complete between two and eight training units per week with an average duration of 90 min per session. The study was conducted in the middle of the season between January and March. Of the 91 participants, 88% (80 participants) throw with the right arm and 12% (11 participants) with the left arm.

The players are divided into the following positions: 22 pivot players (group 1), 9 goalkeepers (group 2), 34 backcourt players (group 3), 27 wing players (group 4).

Only male individuals without any acute diseases in need of treatment such as acute infections or injuries as well as pain in the musculoskeletal system at the time of the measurement were considered to participate in this study. Accordingly, all participants are subjectively healthy or fit for training at the measuring time. Furthermore, a playing experience of up to 10 years is necessary. Serious injuries should date back for at least 1 year. Participation was voluntary.

All subjects were informed about the study design before giving written informed consent. The study was approved by the local medical ethics committee of the medical faculty (No. 518/15).

### Measurement systems

Three dimensional back scan: A three-dimensional back scan was performed to quantify the upper back posture while standing, using the back scan system “ABW-BodyMapper” (ABW GmbH, Frickenhausen/ Germany).

During a measurement sequence 15 photos were taken. The maximum picture frequency of the scanner is more than 50 frames/sec with a spatial resolution of 1/100 mm. One measurement lasts approx. Two seconds. The system error is specified as <1 mm (manufacturer information), the reproducibility is limited by the calculations of the upper body posture defined by markers directly on the skin (<0.5 mm).

**Fig. 1:**
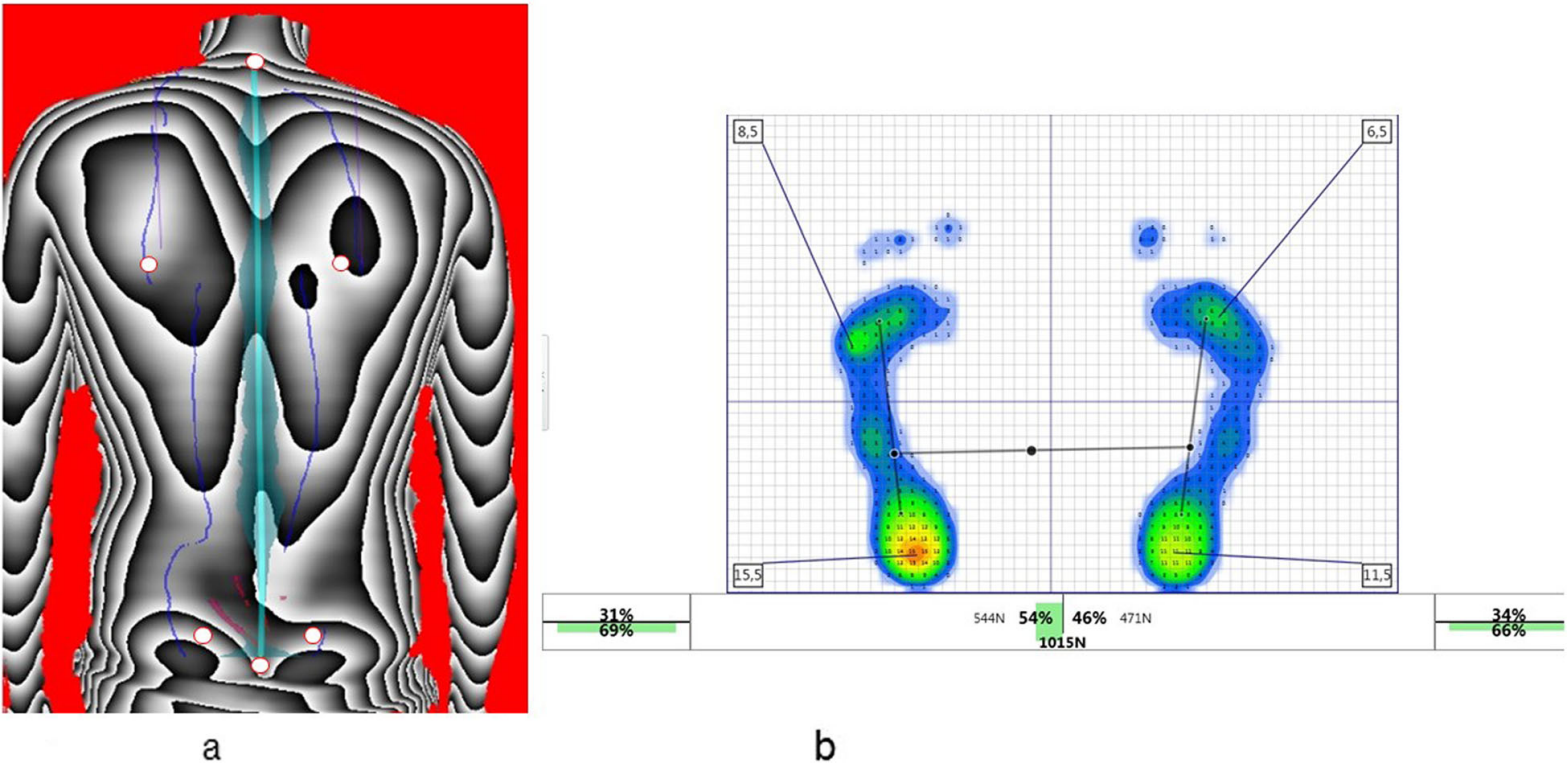
**a** Phase image of the three dimensional back scan. The white dots with the red border mark six antaomic landmarks: Spinal area (markers on C7 and L5), shoulder area (markers at the top of the left/right scapula) and pelvis area (markers on the left/right spina iliaca posterior superior [SIPS]). **b** Representation of the postural control. The percentage distribution of balance and force between the left and right sides of the body as well as the percentage distribution between forefoot and rear foot can be seen. **b** Exemplary representation of the plantar pressure distribution while standing with the pressure measuring plate

For analyzing the phase pictures all test persons were marked at six defined, standardized anatomical locations indicating underlying bone structures (Fig. 1a).

Pressure Measuring Platform: The pressure measuring plate FDM–S (Zebris medical GmbH/Isny, Germany) is used. The plate is 158 cm × 60,5 cm in size with a measuring surface of 149 cm × 54,2 cm. The sampling frequency is 200 Hz, wherein the pressure in the measurement unit “pascal” (force / unit area) is measured (Fig. 1b). According to the manufacturer, the sensors operate with a measurement accuracy of ±5% and a delay rate of ≤3%. The duration of each measurement was 30 s.

### Evaluation parameters

Three dimensional back scan: The three-dimensional back scan was split into three components to quantify the following parameters: spinal area (markers on Cervical vertebra (C) 7 and Lumbar vertebra (L) 5), shoulder area (markers at the top of the left/right scapula) and pelvis area (markers on the left/right spina iliaca posterior superior [SIPS]) (Fig. 1a) [19]. The sign indicates whether it is either a right- or left-sided or anterior or posterior deviation of the respective parameter.

Pressure Measuring Platform: The balance (% weight distribution) between the right and left foot as well as between the forefoot (left and right) and the rear foot is determined. Furthermore, the total force (N) of both feet and the total force per foot are recorded. With regard to the maximum pressure (N/cm^2^), statements can be made for both feet together, the left/right foot as well as the forefoot (left and right) and the rear foot (left and right) (Fig. 1b). The covered area per foot (cm^2^) is the area of the feet as the sum of the area of the sensor elements (maximum pressure), where the pressure is ≠ 0. Accordingly, the total covered area is the sum of both feet. Additionally, data of the ellipse (large and small half axis (mm), angle (°), area (mm^2^) and the length of the Center of Pressure (CoP) (mm) are used. This is the total length of the center of gravity about the recorded measuring sequence.

### Data collection

All measurements will be performed in dimmed, quite rooms with comfortable room temperature.

Participants will be asked to stand barefoot (habitual foot position) on the platform with upper body undressed in habitual posture. The distance between the scanner and the subject is approximately 90 cm. Arms will hang down loosely with the view fixed at a point on the opposite wall on eye level. One measurement lasts for 5 s and will be repeated five times with short periods of rest in between.

### Data analysis

The recorded data was analyzed using BiAS software (version 10.12, epsilon-Verlag; Hochheim, Darmstadt, Germany). The collected parameters were expressed as median, tolerance range and the 95%-confidence interval (95%-CI) of the median. The assumption of normality was tested using the Kolmogorov-Smirnoff-Lilliefors-Test. As the data was not normally distributed the Kruskal-Wallis-Test with following Conover-Iman-Tests for post-hoc-comparisons was used to compare continuous variables between groups. Further, two-sample comparisons were analyzed by the Wilcoxon-Mann-Whitney-U-test or Wilcoxon-Matched-Pairs test with Bonferroni-Holm-corrections and correlations by Spearman’s rank-correlation. All tests were two-tailed and a *p*-value of less than 0.05 was considered as significant.

## Results

### Constitutional parameter

The constitutional parameters body height, body weight and BMI were not normally distributed. The median of body weight was 85.0 kg (tolerance range: 60.6–119.6 kg; 95%-CI:81.0–87.0 kg). For the body height a median of 1.85 m was calculated with a tolerance range between 1.70 and 1.95 m and a 95%-confidence interval of 1.83 to 1.87 m. For the BMI a median of 24.7 kg/m^2^ was calculated, with a corresponding tolerance range from 20.1 to 34.9 kg/m^2^, and a 95%-confidence interval from 24.2 to 25.1 kg/m^2^.

### Upper body posture

From the back scan readings, the posture of an average male handball player was calculated. Table 1 shows the mean, standard deviation, median, first and third quartile as well as the tolerance range (upper/lower limit) and the confidence interval (lower/upper limit) of the upper body statics, which are divided into spinal, shoulder and pelvic parameters.

**Table 1:** Mean, standard deviation, median, 1. and 3. Quartil, tolerance regions (upper and lower limit), 95%-confidence interval (lower and upper limit) of the upper body posture and the postural control

Evaluation parameter	Mean	Standard Deviation	Median	1.quartil	3.quartil	tolerance range lower limit	tolerance range upper limit	confidence interval lower limit	confidence interval upper limit
Upper body posture									
Spine parameter									
Trunk length D (mm)	505.98	26.14	508.13	490.46	524.15	452.97	551.34	500.56	511.39
Trunk length S (mm)	546.42	27.97	546.00	529.48	565.98	498.25	599.30	540.63	552.21
Sagittal trunk decline (°)	−4.22	2.07	− 4.00	−5.70	−3.00	−8.75	−0.58	−4.65	− 3.79
Frontal trunk decline (°)	−0.65	1.07	−0.80	−1.28	0.00	−3.00	1.97	−0.87	−0.43
Axis decline (°)	−0.40	1.85	−0.40	−1.53	0.60	−4.00	3.80	−0.78	−0.01
Thoracic bending angle (°)	15.91	3.23	16.00	14.22	18.05	8.66	21.53	15.24	16.58
Lumbar bending angle (°)	9.78	2.96	9.40	7.68	11.37	5.19	16.98	9.18	10.41
Standard deviation lateral deviation (mm)	4.85	2.36	4.40	3.21	6.20	1.37	11.35	4.37	5.34
Standard deviation rotation (°)	3.77	1.65	3.45	2.40	4.85	1.62	8.59	3.43	4.11
Kyphosis angle (°)	47.04	8.27	47.25	41.11	52.95	30.94	63.99	45.33	48.76
Lordosis angle (°)	35.57	10.29	35.00	28.91	42.55	15.05	57.95	33.44	37.70
Shoulder parameter									
Scapular distance (mm)	198.93	22.93	200.13	181.95	216.20	159.20	240.79	194.78	203.67
Scapular height (°)	−3.61	8.19	−4.50	−8.47	1.84	−19.43	12.37	−5.03	−1.91
Scapular rotation (°)	1.08	2.74	1.32	−0.69	3.00	−5.51	6.14	0.51	1.64
Scapular angle left (°)	25.95	5.55	26.20	22.30	29.00	15.40	39.60	24.80	27.09
Scapula angle right (°)	27.85	6.23	27.93	24.85	31.56	9.82	41.19	26.56	29.14
Pelvis parameter									
Pelvis distance (mm)	111.17	19.23	111.70	95.13	124.56	76.60	153.62	107.19	115.15
Pelvis height (°)	0.22	1.60	0.00	−1.00	1.00	−2.78	4.48	−0.11	0.55
Pelvis torsion (°)	0.65	4.60	0.67	−1.71	2.60	−8.22	12.27	−0.31	1.60
Pelvis rotation (°)	−0.78	3.05	−0.75	−2.60	1.40	−7.23	5.61	−1.41	−0.15
Postural control									
Percentage body weight distribution (%)									
Left	49.45	4.35	49.37	47.00	52.40	41.35	58.65	48.55	50.35
Right	50.55	4.34	50.63	47.60	53.00	41.35	58.65	49.64	51.44
Forefoot left	36.95	9.43	37.09	30.63	43.18	15.62	54.89	34.99	38.90
Forefoot right	38.33	11.48	37.10	31.98	44.05	18.99	65.32	35.95	40.71
Rearfoot left	62.51	8.94	62.66	56.17	68.97	44.98	82.06	60.65	64.36
Rearfoot right	60.59	11.29	62.53	54.95	67.66	32.08	79.59	58.27	62.91
Force parameter (N)									
Total force	816.50	133.57	804.60	726.45	908.75	602.68	1167.83	788.84	844.16
Force left foot	404.00	81.65	393.54	345.43	457.19	276.90	621.81	387.09	420.92
Force right foot	412.50	67.53	407.16	366.71	457.00	295.92	540.75	398.51	426.49
Pressure parameter (N/cm^2^)									
Maximal pressure	14.21	3.88	13.45	11.28	16.00	8.04	23.21	13.40	15.01
Left foot	13.24	4.05	12.80	10.21	15.33	6.13	22.14	12.40	14.08
Right foot	12.61	3.25	12.36	10.67	14.00	7.27	20.81	11.94	13.28
Forefoot left	6.85	2.42	6.09	5.20	8.00	4.07	13.90	6.34	7.34
Forefoot right	7.21	2.67	6.60	5.17	8.40	4.00	14.27	6.66	7.76
Rearfoot left	12.97	4.19	12.61	10.18	15.33	6.13	22.14	12.10	13.84
Rearfoot right	12.03	3.58	12.00	9.21	13.90	5.83	20.81	11.29	12.77
Covered area (cm^2^)									
Covered area left foot	107.28	16.31	107.10	93.86	120.69	77.04	143.67	103.90	110.67
Covered area right foot	111.43	15.07	112.11	99.85	123.13	85.44	142.61	108.31	114.55
Total covered area	218.61	30.24	217.40	191.90	241.55	165.76	284.34	212.35	224.88
Ellipse									
Large half axis (mm)	2.59	1.24	2.25	1.81	3.00	1.20	5.41	2.32	2.84
Small axis (mm)	0.81	0.38	0.80	0.58	1.00	0.00	1.60	0.73	0.89
Angle (°)	0.69	19.65	−0.61	−14.09	14.95	−33.13	51.70	−3.38	4.75
Area (mm^2^)	7.93	10.27	5.39	3.76	8.75	2.00	22.25	5.81	10.06
CoP (Centre of Pressure)									
Lenght (mm)	26.08	4.29	25.00	23.25	28.00	20.60	37.14	25.20	16.97

On average the subjects are slightly inclined in posterior line of −4.00° (tolerance range: 8.75° ventrally to 0.58° dorsally; 95%-CI: −4.65- -3.79°). Laterally a minimal deviation of 0.80° to the left of the frontal trunk decline was seen (tolerance range: − 3.00- 1.97°; 95%-CI: − 0.87– − 0.43°). The axial deviation (as lateral inclination between upper body and pelvis) was in the mean value slightly tilted to the left (0.40°) with a tolerance range of approx. ±3.5° and a 95%-confidence interval of <1° (− 0.78° respectively − 0.01°). This implied that there are no obvious differences in the lateral inclination between the upper and lower body (Table 1).

The thoracic bending angle was calculated from the distance between the vertebra prominens and the kyphosis apex and indicated the deviation from the perpendicular line. The median angle was 16.00° confirming the expected thoracic kyphosis. Here, wider variations were indicated by a tolerance value of 8.66 and 21.53° and a 95%-confidence interval of 15.24and 16.58°. The lumbar bending angle has a mean of 9.40° (tolerance range: 5.19°-16.98°; 95%-CI: 9.18–10.41°). The lumbar bending angle describes the deviation of the distance between the lordosis- and kyphosis apex. The kyphosis and lordosis angle have a median of 47.25° and 35.00°, with in a substantial tolerance range of 30.49° and 63.99° for the kyphosis angle and a tolerance range of 15.05° and 57.95°for the lordosis angle. The 95%-confidence interval of both angle is about ±1–2° of the median.

Measurement of the standard deviation of the lateral deviation shows a right-sided inclination of the median line by 4.40° when connecting the points vertebra prominens and the center of the pelvic markers. Both, the tolerance range (1.37° respectively 11.35°) as well as the 95%-confidence interval (4.37°/5.34°) indicate a right-sided deviation. The standard deviation of the rotation of the spinal column is a marker of the spinal torsion considering the spinous processes of the vertebrae. A negative value described a rotation to the left and a positive value to the right. The median rotation was 3.45°, with a tolerance range between 1.62° and 8.59°, and a 95%-confidence interval between 3.43° and 4.11°. Consequently, on average a right sided spinal rotation was found.

The scapula distance values as indicator of the variability of the upper body was 200.13 mm, with a tolerance range of 159.20–240.79 mm, and a 95%-confidence limit of 194.78–203.67 mm. The scapular height (deviation from the horizontal line) refers to shoulder stand where the left shoulder blade is 4.50° more caudally, whereas the lower and upper limit of the tolerance range markers were − 19.43° and 12.37° and the lower and upper limit of the confidence interval is − 5.03° or rather − 1.91°. The tolerance range and the 95%-confidence interval illustrate that the scapular height can vary between or more caudally left or right shoulder marker. There is almost no rotation of the shoulder markers with a median of 1.32° and a tolerance range of − 5.51° to 6.14° and a 95%-confidence interval of 0.51° to 1.64°. Only minor differences between the left and right shoulder blade angle show that the right shoulder is about 2° (median) more caudally.

The pelvic position anchors the body. The distance for the fixed markers on the spina iliaca posterior superior refers to the pelvic width, which is on average 111.70 mm (tolerance range: 76.60–153.62 mm, 95%-CI:107.19–115.15 mm). The deviation of the pelvic height identifies the horizontal plane and deviations from this. The pelvis position is ideally balanced (median: 0.00°). The same applies to the pelvis torsion and rotation, so that the iliac left is marginally rotated posteriorly and simultaneously tilted further ventral (mean pelvis torsion: − 0.75°; Mean pelvic rotation: − 0.75°).

### Postural control

Table 1 also shows the mean, standard deviation, median, tolerance range (upper/lower limit) and 95%-confidence interval (lower/upper limit) of the postural control.

The handball players show a basically balanced weight distribution with 49.37% (tolerance range: 41.35–58.65%; 95%-CI: 48.55–50.35%) on the left and 50.63% (tolerance range: 41.36–58.75%; 95%-CI: 49.64–51.44%) on the right foot.

The rear feet are heavier loaded than the forefeet (left foot: 62.66%; right foot: 62:53%). The tolerance range and the 95%-confidence interval also indicate an increased weight load on the rear foot (Table 1). On the right side of the body there is a little more force than on the left (407.16 N: 393.54 N), which remains the same even at maximum pressure. The left body side has a pressure of 12.80 N/cm^2^ and the right body side 12.36 N/cm^2^. The pressure on the rear foot of both sides of the body is twice greater than that of the forefoot (12.61 or 6.09 N/cm^2^: 12.00 or 6.60 N/cm^2^), similar to the percentage weight distribution. This almost ideally balanced postural control is confirmed by the total covered area of both feet (Table 1).

The ellipse ranges between the large half axis with 2.25 mm and the small half axis with 0.80 mm with an area of 5.39mm^2^ (tolerance range: 2.00–22.25mm^2^; 95%-CI:4.80–5.75mm^2^) and an angle of − 0.61° (tolerance range: − 33.14- 51.57°; 95%-CI: − 7.30- 4.60°). Accordingly, the predominant direction of fluctuation is almost from anterior to posterior. The length of the CoP is 25.00 mm (tolerance range: 20.60–37.14 mm; 95%-CI: 24.20–26.43 mm).

Correlations between upper body posture/postural control and BMI and playing years.

There is a poor negative correlation between the BMI and the frontal trunk decline (*p* ≤ 0,04; rho: − 0,22) and no correlations (*p* ≥ 0.05) between the BMI and the the postural control parameters. Correlations between the years of playing and the upper body posture can only be found at the pelvis height (*p* ≤ 0.04; rho: − 0.21) and for the length of the CoP (*p* ≤ 0.01; rho: −2.09).

### Comparisons

#### Left vs. right throwing arm

A comparison of the left and right throwing arm regarding the parameters of the upper body posture and the parameters of the postural control reveals no significant differences (*p* ≥ 0.05) after Bonferroni-Holm correction.

#### Playing position

**Fig. 2:**
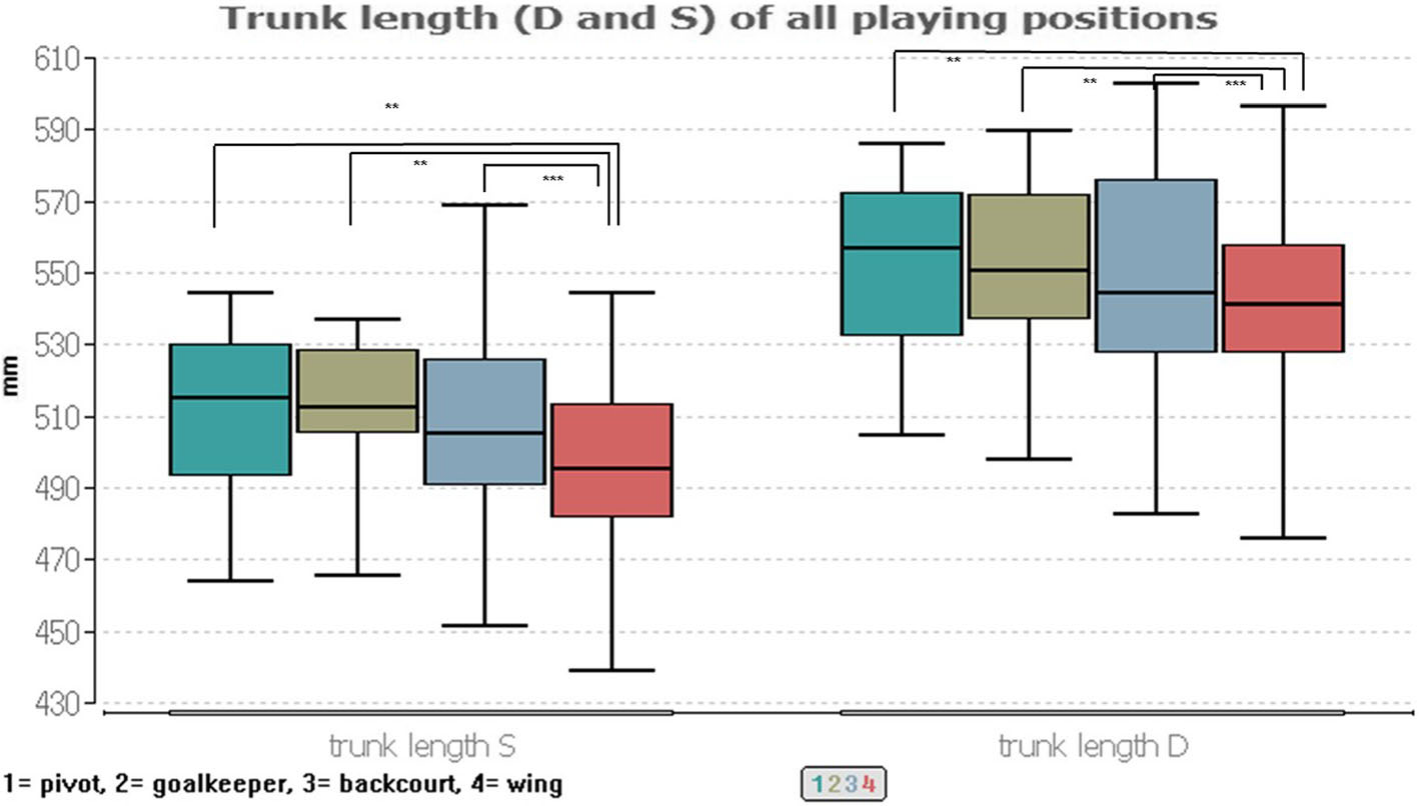
Trunk length (D and S) of all playing positions

The body height of *p* ≤ 0.001 (Kurskal-Wallis test) is significantly different between the game positions. The following Conover-Iman-Comparison in line with a Bonferroni-Holm correction shows significant differences between pivot player and wing player (*p* ≤ 0.03), goalkeeper and wing player (*p* ≤ 0.03) and backcourt player and wing player (*p* ≤ 0.001). These differences are due to the smaller wing players between 6.5 and 8.5 cm compared to the players in the other positions (Fig. 2).

The playing position seems to be independently of the BMI, age or the upper body posture, since no significant group differences (*p* ≥ 0.05) were calculated here.

**Fig. 3:**
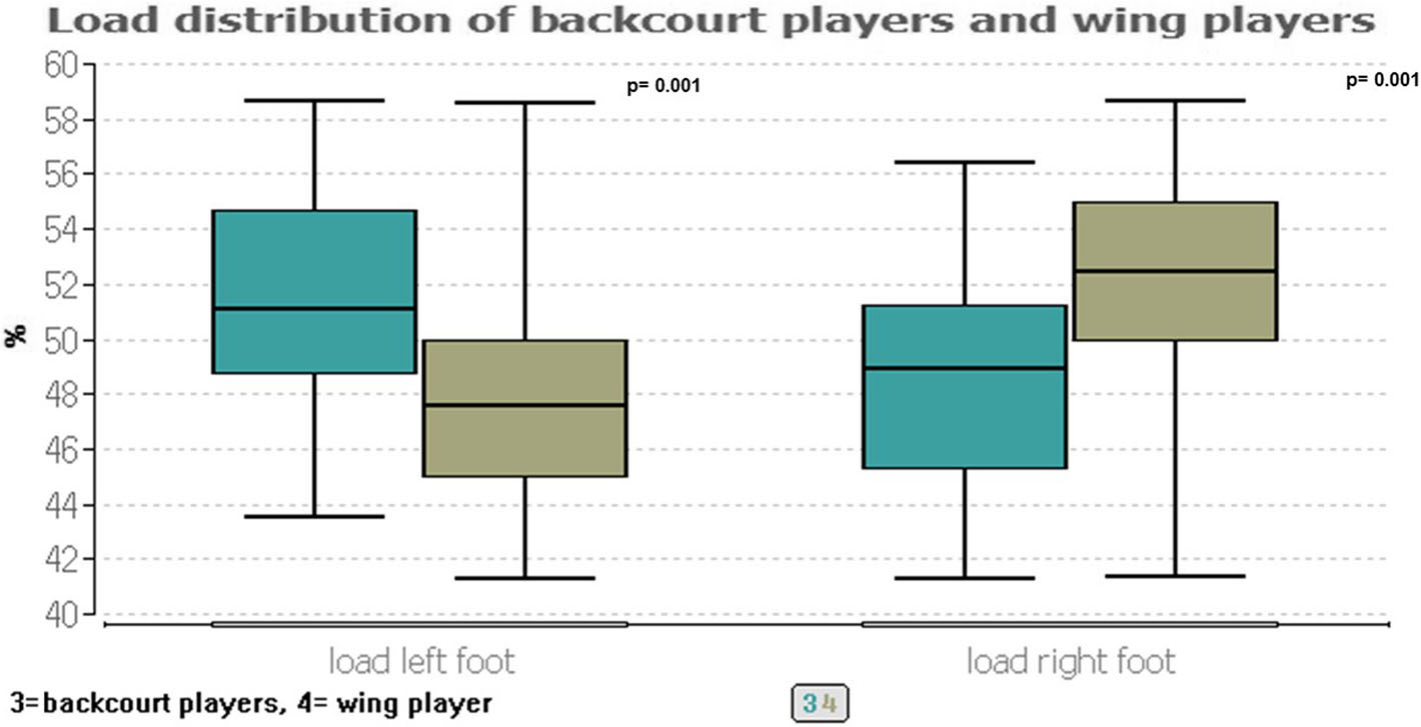
Load distribution of Backcourt players and wing players

In contrast, a significant group difference of *p* ≤ 0.001 can be seen with the percentage load of the left foot, after which backcourt players have a higher load than wing players (51.1: 47.6%; *p* ≤ 0.001) (Fig. 3). Accordingly, the right foot is significant loaded more in wing players (52.40%) than in backcourt players (48.90%) (*p* ≤ 0.001). The percentage distribution in the forefoot and rear foot area of both feet remains unchanged like all other parameter (the total force, maximum, covered area, ellipse (large and small half axis), ellipse angle, ellipse area, length of the CoP).

With regard to the left-right comparison of the parameters of postural control in the individual play positions (Wilcoxon-Matched-Pairs test), the following significance can be noted:

- *Pivot player*: Pivot players have only a significant difference in the covered area (*p* ≤ 0.01), according to which the right area (112.55 cm^2^) is larger than the left area (111.40 cm^2^).
- *Goalkeeper*: Goalkeeper show no differences in the left-right comparison of postural control.
- *Backcourt player*: Backcourt player have a significant different weight distribution between the left and right rearfoot (*p* ≤ 0.01), whereas the median shows only marginal difference of 62.52% (left) and 62.30% (right).
- *Wing player*: The right foot is more heavily loaded (52.40%) than the left foot (47.60%) (*p* ≤ 0.001) which can be supported by the force distribution (*p* ≤ 0.001), where the right foot (415.60 N) also has a higher force load than the left foot (374.80 N). In addition, the covered area of the right foot is significantly larger (*p* ≤ 0.001; left: 108.33cm^2^, right: 116.67cm^2^).

## Discussion

This study was conducted to analyze the relationship between playing position, playing years, BMI or throwing arm and the upper body posture as well as the postural control in German handball players.

With regard to the constitutional parameters body height, weight and BMI in comparison to persons of the same age and sex from Germany [20–22], it can be seen that German handball players are on average minimally taller and heavier. The variation range of the BMI of these three studies is approx. Between 23 and 26 kg/m^2^, whereby the BMI of male handball players of this study is in the middle of the data with 24.7 kg/m^2^.

**Fig. 4:**
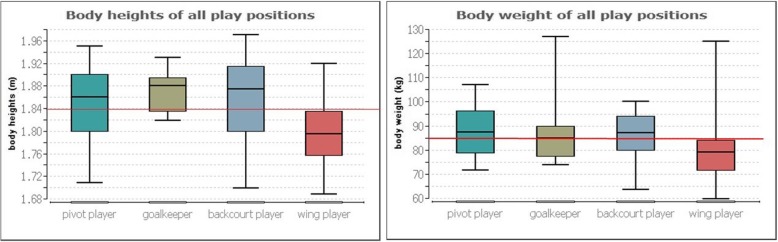
Body heights (left picture) and body weight (right picture) of all play positions

Furthermore, the comparison of the body sizes and the weight between the individual playing positions shows that only the wing players are below the average of all players (1.84 m and 85 kg respectively; red line in Fig. 4).

Consequently, the results of height and weight are identical to those of Povacos et al. [7] and Michalsik et al. [8–10] as far as the constitutional prerequisites are concerned and prove hypothesis 1. Wing players are between 6.5 and 8.5 cm significantly smaller than the other players. However, this cannot be proven in the trunk length, so that here only shorter lower extremities can be inferred in comparison to the other players. In this context, however, it should also be noted that the players played in different leagues. It may also be possible that the height of the players may vary depending on their performance level. As an influencing factor, however, it must also be taken into account that a kind of natural selection is involved through the constitution. Taller athletes have for instance a bigger chance to play handball at higher level.

The comparison of the upper body posture of handball players with those of healthy men between 18 and 30 years [22] also shows that the descriptive parameters (median, tolerance range and confidence interval) are similar. Only the kyphosis angle in the thoracic spine is about 2–5° larger in the median for handball players. Furthermore, it can be stated that the scapular distance is about 20 mm greater. In addition to the assumption that this is due to more muscle mass in the shoulder girdle, this can also be connected with a protraction of the shoulder girdle. The pelvis distance, which is about 17 mm (median) wider, is also conspicuous. Therefore, it could be concluded that the players have a larger shoulder blade distance and a larger iliacal distance and thus have a “stronger constitution”. However, this assumption should be analyzed in further studies. With regard to postural control, Pomarino & Pomarino [23] and Scharnweber et al. [24] confirm the increased percentage weight distribution between the forefoot and rearfoot, whereby the distribution between the two sides of the body is almost balanced. However, the left/right load ratio was found to be more balanced in handball players (50%: 50% distribution;) than in normal healthy individuals (46%:64%) [24]. The very good postural stability (ellipse) of young Polish goalkeepers of the Polish National Junior Handball as presented by Wilczyński [18], are very comparable with the present data of older male handball players.

Available results show that high-intensity, professionally designed handball training in men, which begins in early childhood, does not cause muscular imbalances in the upper body posture or deviations in postural control. Grabara [25, 26] confirms that a two-year handball training does not cause asymmetries of posture in adolescents even in comparison to an aged-matched non-athlete control group. In his opinion, despite asymmetric elements, handball training does not negatively affect the posture in the frontal and transverse planes [26]. Accordingly, based on the results of Póvoas et al. [27], a recreational handball training as a health enhancing intervention could be used, which does not cause lasting muscular imbalances. However, how muscular training develops in people who did not start handball in childhood or who only play leisure time sports cannot be concluded from these findings.

Since there are no correlations between the BMI and the upper body posture and the postural control hypothesis 2 can be falsified. The same can be confirmed for the age factor. However, there is a negative correlation between the years of playing and the pelvis heights to the effect that the right pelvic heightening changes with increasing years of play towards a higher left pelvic heightening. However, the amount or extent (independently from the elevated body side) of the pelvic elevation remains predominantly between 0 and 6°. Furthermore, the length of the CoP decreases with increasing playing experience. A balanced weight distribution or rather a good balance is a coordination ability and desirable regarding to the necessity of efficient responses in constantly changing play situations. The length of the CoP of the present handball players is mainly between 20 and 37 mm and the age of most of the test persons is between 16 and 30 years, from which the playing experience can also be calculated back, since mostly in (early) childhood a sport is started. In this context, the playing level and thus the number of weekly training sessions seems to have a more significant influence on the playing experience than the number of matches. The physiological mechanisms that may lead to the result that regular, intensive handball training shortens the length of the CoP should be further investigated. Based on these results, hypothesis 3 cannot be confirmed. A more asymmetric posture and weight distribution has not occurred with increasing years of playing.

Furthermore, the comparison of the left and right throwing arm reveals no significant differences in terms of upper body posture and postural control. Consequently, there is no evidence to support handiness related changes in the muscle development of the dorsal back muscles and the sensorimotor control of the balance regulation (hypothesis 4). In this context, however, the distribution of left (12%) and right (88%) handed players must be taken into account. A further analysis with equivalent throwing arm distribution would provide further insights in this respect.

Although in handball different physiological demands for different playing positions are required and backcourt players or pivot players are taller and heavier as well as show higher intensities (max. Heart rate) than wing players (left/right side of the game), these physiological demands are not recognizable in different upper body posture and only slightly in postural control [7–10]. Wing players are therefore the smallest handball players and have the most asymmetric load distribution between the two sides of the body, whereby this asymmetry is within 5% and therefore cannot really be considered clinically relevant. Only the increased length of the CoP can be considered clinically relevant, as it is a median difference of about 8 mm. Here the cause should also be investigated in further analyses. Hypothesis 5 should therefore be confirmed.

The playing positions show that pivot player, goalkeeper and backcourt player do not differ constitutionally (body height, weight and BMI) as well as with regard to their upper body posture and balance. Only wing players are smaller and heavier. Since goalkeepers do not differ from pivot players or backcourt players, this can be traced back to the training, which is done the same way by all. Offensive and defence within a 60-min, high-intense technical handball game with strength-related playing actions and high-intensity changes seems to presuppose this very requirement of a physical constitution, which is reflected above all in the body size and weight [1, 2] but also presupposes symmetries of the upper body posture and balance distribution. Whether the same results can be proven for basketball players, for example, according to their playing position, will have to be investigated in the future. In this context, Oyama et al. [28] demonstrate differences between the dominant and non-dominant scapula side in healthy overhead athletes (baseball pitchers, volleyball players, and tennis players) through the use of an electromagnetic tracking device. Therefore, they emphasize the importance of accurately assessing pathologic changes in bilateral scapular positions and orientations after injury [28].

Sanchis-Moysi et al. [29] could prove in tennis players differences in muscle volume, fiber-type distribution and muscle strength in the upper extremity between the dominant and the non-dominant arm. The same applies to bone density in tennis players [30]. Such findings for handball players do not yet exist, but should definitely be researched in the context of current framework training plans.

The present study confirms the results of previous investigations (with a lower number of test persons) with regard to the constitutional differences of the players in the various play positions and with regard to a symmetrical upper body posture. The present data therefore confirm and complete the already existing data, because the upper body posture and the postural control have not been analyzed simultaneously on the same collective to date. This verifies that the dynamic, variable sport of handball causes a balanced upper body posture of the athletes, which is also reflected in a balanced postural control.

Thus, the present data could be used as reference values for upper body posture or postural control for active handball players. Since the risk of injury in handball is very high, especially in the shoulder or lower extremity area, data of inactive handball players due to injury could be compared with the available data in the context of therapy induction or follow-up of (sports) rehabilitation.

## Conclusion

It can be concluded that handball players on a professional level, due to the quality of the training, show neither asymmetries of the upper body posture nor of the load distribution (postural control). The throwing arm and the corresponding muscular characteristics on this side do not seem to be a factor of influence either, since a handball throw requires a high degree of rapid strength, which does not require much muscle mass, since the throwing device is very light and a correspondingly rapid increase in strength is mainly due to inter- and intramuscular coordination mechanisms. There are also no differences in playing experience. To maintain symmetric upper body statics and balanced postural control, regular and intensive handball training for men is recommended.

## Data Availability

The datasets used and/or analyzed during the current study are available from the corresponding author on reasonable request.

## Abbreviations

BMI: Body Mass Index
C: Cervical vertebra
CI: Confidence interval
CoP: Center of Pressure
HBL: “Handball Bundesliga”
L: Lumbar vertebra
SIPS: Spina iliaca posterior superior
